# Study protocol: the SPInal NAVigation (SPINAV) trial – comparison of augmented reality surgical navigation, conventional image-guided navigation, and free-hand technique for pedicle screw placement in spinal deformity surgery

**DOI:** 10.1186/s12891-025-08817-3

**Published:** 2025-06-02

**Authors:** Victor Gabriel El-Hajj, Anastasios Charalampidis, Daniel Fell, Erik Edström, Adrian Elmi-Terander, Paul Gerdhem

**Affiliations:** 1 Department of Clinical Neuroscience, Karolinska Institutet, Stockholm, Sweden; 2 Department of Clinical Science, Intervention and Technology (CLINTEC), Karolinska Institutet, Stockholm, Sweden; 3 Department of Reconstructive Orthopedics, Karolinska University Hospital, Stockholm, Sweden; 4 Capio Stockholm Spine Center, Löwenströmska Hospital, Box 2074, Stockholm Upplands-Väsby, 194 02 Sweden; 5 Department of Surgical Sciences, Uppsala University, Uppsala, Sweden; 6 Department of Orthopedics and Hand Surgery, Uppsala University Hospital, Uppsala, Sweden

**Keywords:** Navigation, Spinal instrumentation, Pedicle screws, Augmented reality

## Abstract

**Background and purpose:**

Although navigation is increasingly used in spinal surgery, the advantage of different navigation technologies is still a matter of debate. Conventional image-guided navigation is currently the gold standard. However, modern, Augmented reality-based navigation methods are increasingly gaining ground. Surgical navigation in deformity surgery allows placement of pedicle screws in small and deformed pedicles and may result in both a higher accuracy and density of pedicle screw placement. The aim of this trial is to compare AR and conventional surgical navigation to free-hand technique.

**Patients and methods:**

This is a single center, open label, parallel assignment, three arm, randomized, controlled trial, comparing: Augmented reality surgical navigation (ARSN), Infrared surgical navigation (IRSN) and Free-hand (FH) technique. Individuals scheduled for spinal deformity surgery are eligible for inclusion. The inclusion criteria are written informed consent, age ≥ 12 years and spinal deformity. Subjects will be randomized intraoperatively and strictly sequentially.

**Outcomes:**

The primary endpoint is accurately placed pedicle screws based on intraoperative verification cone beam computed tomography (CBCT) scan. All radiological image analyses, on both intra- and postoperative imaging will be performed postoperatively by blinded reviewers.

Several secondary outcome measures including revision rate, radiation exposure, implant density and final accuracy will be analyzed. Patient reported outcomes will also be assessed. Finally, a cost–benefit analysis will be performed.

**Start of trial and estimated duration:**

The SPINAV trial started recruiting patients in January 2022 and will continue for approximately 2.5 years.

**Trial registration:**

The trial is registered at clinicaltrials.gov (NCT05107310) on 2021–11-03.

## Introduction

Spinal deformity constitutes a prevalent indication for surgery in childhood, with approximately 17 out of 100,000 individuals aged 0 to 18 undergoing spinal deformity surgery between 2015 and 2018, as indicated by a nationwide UK-based study [1]. Furthermore, the incidence of spinal deformity surgery is increasing in older adults, evident in a nationwide US registry reporting a noteworthy 141% surge in the utilization of long-construct fusions [2].

The complexity of spinal deformity surgery is underscored by its narrow error margins. Moreover, precise placement of pedicle screws and implants in severely deformed spines is particularly challenging. The pedicle's narrow dimensions magnify the importance of accuracy, as misplaced screws can lead to severe vascular, pulmonary, or neural injuries, along with compromised bone purchase [3].

While the reported accuracies of freehand pedicle screw placement in the thoracic and lumbar spine are highly variable, the benefits of improved accuracy, especially in the context of minimally invasive surgery, are evident.

Intraoperative fluoroscopy has been the traditional image guidance method for pedicle screw placement, offering a two-dimensional imaging perspective. The constant development of three-dimensional (3D) imaging technologies for intraoperative use has paved the way for surgical navigation within the field of spine surgery [4].

Computer-assisted navigation using intraoperative 3D imaging has been shown to improve screw placement accuracy, reduce complications due to screw misplacements, and consequently reduce the frequency of postoperative revision surgeries, when compared to conventional free-hand technique [5, 6].

In the context of spinal deformity surgery, previous reports have underscored enhanced accuracies when employing surgical navigation in scoliosis surgery, particularly in cases where anatomical intricacies pose heightened surgical challenges [7, 8].

Most of the commercially available navigation systems are based on infrared cameras, using outside-in detection of reflective spheres on a dynamic reference frame fixed to the patient’s spine. Emerging video-based systems incorporating augmented reality (AR) for surgical navigation, along with intraoperative three-dimensional imaging, represent innovative computer-assisted navigation techniques [9–11]. These systems have demonstrated relatively high accuracy, safety, and effectiveness in previous studies reports on pedicle screw placement [4, 12, 13].

Nonetheless, as of now, the majority of data in this area is based on retrospective and a few prospectively collected series [12, 14, 15], while randomized controlled trials on spinal deformity are lacking. This is similar to the current situation of evidence on robotic pedicle screw placement, where the evidence from randomized studies comparing robotic guidance to navigation is scarce [6, 16, 17].

The aim of this trial will hence be to compare conventional and AR surgical navigation to free-hand technique based on three main study domains: implant placement accuracy, intraoperative radiation exposure and metrics, and operative time for navigation. Intraoperative adverse events will also be reported. Finally, since data on cost-effectiveness is crucial to ensure adequate allocation of healthcare resources, the cost-effectiveness of this technology will be assessed.

## Methods and analysis

### Overview of study design

**Fig. 1:**
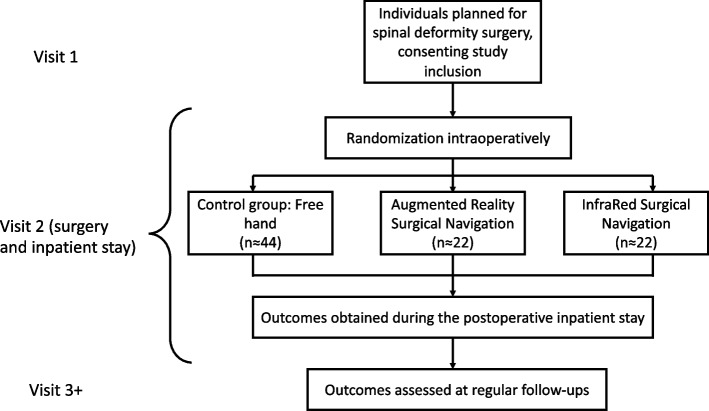
Flowchart describing the study design

This is a single center open label parallel assignment three arm randomized controlled trial (Fig. 1). The trial will use a pragmatic approach and use existing equipment and document handling at the Karolinska University hospital and the existing quality registry for spine surgery, Swespine, for outcome assessments (www.swespine.se).

Study arms:

1. Navigation group 1: Augmented reality surgical navigation (ARSN)
2. Navigation group 2: Infrared surgical navigation (IRSN)
3. Control group: Free hand (FH)

#### Study center and surgeon expertise

The Karolinska University Hospital is a publicly funded tertiary care center serving a region of approximately 2 million inhabitants. It is the region’s only spinal deformity center and handles all deformity surgeries in the region, with approximately 100 spine deformity surgeries being performed yearly. The study uses a pragmatic approach and use already available infrastructures, such as already existing navigation systems, hospital registries, radiology archiving and communications system, and the Swespine registry.

All surgeries were performed by senior orthopedic spine surgeons with at least 10 years of experience.

## Patient selection

### Subject enrollment

Individuals scheduled for spinal deformity surgery will be informed of the study at the time of an outpatient visit at the Karolinska University hospital.

Subject eligibility will be established before treatment randomization. Subjects will be randomized intraoperatively and strictly sequentially, as subjects are eligible for randomization. If a subject discontinues from the study, the subject number will not be reused, and the subject will not be allowed to re-enter the study. The inclusion will end when the sample size has been reached in both intervention arms.

### Inclusion criteria

• Written informed consent.

• Age 12 years and older.

• Spinal deformity surgery.

### Exclusion criteria

• Unable to give informed consent.

• Surgery without pedicle screws.

• Previous surgery with pedicle screws in the planned surgical area.

### Discontinuation and withdrawal of subjects

Subjects are free to discontinue their participation in the study at any time. This will not affect further treatment. Already collected study data for these patients will be kept in the study database, however new data, including data collected from registries will not be added.

### Premature termination of the study

The study group may decide to stop the trial or part of the trial at any time. In case of premature termination, the investigators will promptly inform the Ethics Committee and provide a detailed written explanation, which will be made publicly available.

### Randomization and blinding

The subjects are randomized in a 1:1 ratio, with stratification for operating theatre since the IRSN or ARSN system are located in different operating theatres. Randomization will take place intraoperatively after completed surgical exposure of the posterior elements and will be performed through the web-based platform of the Swespine registry using an allocation sequence hidden from the healthcare staff. Block randomization will be used. Block sizes will be unknown for the researchers.

The study design does not allow blinding of the surgeon. Since the primary outcome is assessed at the time of surgery and a short time thereafter, we do not find it necessary to blind the patient.

Intraoperatively, CBCT verification scans will be assessed by the surgeon to make decisions on screw revision. However, all radiological image analyses, on both intra- and postoperative imaging will be performed postoperatively by blinded reviewers.

Patient reported outcomes will however be assessed without influence of the care giver.

### Study interventions and standard of care

#### Surgical treatment

All treatments involve surgical stabilization and fusion of the deformed spine. Additional surgical treatments for correction, such as osteotomies are performed as deemed by the treating surgeon.

All forms of treatment are available and used today at the Karolinska University hospital. The choice of supplier and brand of spine implants, as well as the use of intraoperative ARSN or IRSN are based on the preference of each surgeon. Neurophysiological monitoring is always performed as routine during spinal deformity surgery, with the exception of patients that are non-ambulatory. Motor evoked potential, sensory evoked potential and electromyography data will be collected to investigate any changes in neurophysiological parameters. Any deviations will be noted. Electromyography thresholds for all pedicle screws will be recorded.

Regardless of inclusion or randomization, patients will receive routine postoperative care including rehabilitation at the study center.

#### Augmented reality surgical navigation

After standard exposure of the region of interest of the spine a cone beam CT (CBCT) (Philips Allura, Philips, Best, The Netherlands) of the area is performed. Navigation is based on the recognition of optical markers (fiducials) on the skin by the equipment during the CBCT and navigation [12, 18, 19]. The CBCT will result in a three-dimensional image of the spine in which screws are placed virtually on a computer screen. Navigation then takes place. The navigation will show the entry point and direction of the planned screws on an augmented reality image screen in the operating theatre [14, 20, 21]. After placement of the screws, the positioning is checked with a second CBCT. Misplaced screws may be repositioned or extracted.

After screw placement, rods are placed, correction of the deformity and fusion are performed, and the wound is closed.

### Infrared surgical navigation

After standard exposure of the region of interest of the spine a CBCT of the area is performed using the O-arm (Medtronic, Minneapolis, Minnesota, USA) and Universal Air (Brainlab spinal navigation system, Brainlab AG, Munich, Germany). Navigation is based on the recognition of optical markers on a dynamic reference frame attached to a bony prominence of the spine (Brainlab). Navigation requires detection of the optical markers through infrared light and that the reference frame is tightly fixed to the spine. The CBCT will result in a three-dimensional image of the spine in which screws are placed virtually on a computer screen. After placement of the screws, the positioning is checked with a second CBCT. Misplaced screws may be repositioned or extracted [22].

After screw placement, rods are placed, correction of the deformity and fusion are performed, and the wound is closed.

### Free hand

After standard exposure of the region of interest of the spine screws are placed based on anatomical landmarks and feeling of intact bony canals made in the pedicle [14]. If needed, fluoroscopy is performed to check the planned screw paths. After placement of the screws, the positioning is checked with a cone beam CT (O-arm or Philips Allura). Misplaced screws may be repositioned or extracted. After screw placement, rods are placed, correction of the deformity and fusion are performed, and the wound is closed.

### Study outcomes and variables

Primary and secondary study outcomes will be studied in comparisons between ARSN and free hand, IRSN and free hand, and ARSN and IRSN.

#### Primary outcomes

The primary endpoint is accurately placed pedicle screws based on intraoperative verification scan by cone beam computed tomography (CBCT).

An accurately placed screw is defined as one that is placed with less than 2 mm cortical breach of the pedicle (corresponding to Gertzbein grade 0–1) [23].

The endpoint, accurately placed pedicle screws, is calculated as: the number of accurately placed screws (Gertzbein grade 0 + 1) according to intraoperative verification CBCT scan divided by the total number of placed screws.

Screws that are not placed and screws that are placed but repositioned before intraoperative verification CBCT scan are noted in the Case report form (CRF).

#### Secondary outcomes

The secondary objectives of this study are to evaluate accuracy, implant density, radiation exposure and various other measures of surgical and postsurgical success.

#### Secondary outcomes related to accuracy

- Pedicle screw intraoperative revision rates
  - Intraoperatively revised based on clinical assessment.
  - Intraoperatively revised based on neurophysiology.
  - Intraoperatively revised based on intraoperative verification CBCT scan.
- Technical accuracy for ARSN and IRSN, defined as:
  - Deviation from planned navigation path in mm at bone entry and screw tip measured on the postoperative computed tomography (CT).
  - Angular deviation of the placed screw compared to planned navigation path measured on the postoperative CT.
- Accuracy at 1 st attempt = (screws placed at first attempt according to intraoperative protocols and graded 0 or 1)/(total number of placed screws). Assessed on intraoperative CBCT.
- The secondary endpoint, Final accuracy of placed pedicle screws, is calculated as: number of accurately placed screws (Gertzbein grade 0 or 1) according to postoperative CT/total number of placed screws.

#### Secondary outcomes related to implant density

- Pedicle screw placement density comparison. The study aims for 100% pedicle screw density. Hooks may be placed as rescue or if screw placement fails.
- Final implant density compared to planned 100% pedicle screw density.
- Pedicle screw density.
- Hook density.
- Morphometric measurement for regression analysis.
- Pedicle diameters are measured on preop CT.
- Deformity correction rate.
- Cobb angle improvement.

#### Secondary outcomes related to radiation dose exposure

The following objectives will be measured by estimates from the CBCT systems in the operating theatres (estimated by dose area product/kerma area product, radiation time, computed tomography dose index) and dosimeters.

- Patient radiation exposure (ED in mSv) for the whole procedure.
- Patient radiation exposure (ED in mSv) for fluoroscopy.
- Patient radiation exposure (ED in mSv) for each CBCT.
- Average staff radiation exposure (in Sv) for the whole procedure.

#### Secondary outcomes related to surgical and postsurgical parameters

- Patient reported outcome measures:
  o The SRS-22r is a scoliosis specific questionnaire aiming to estimate quality of life in patients with scoliosis [24]. It contains 24 items divided into 5 domains covering function, pain, self-image, mental health, and satisfaction. An index value is calculated for each domain and a total index value is possible to calculate ranging from 1 (worst) to 5 (best). SRS-22r will be collected from Swespine.
  o EOSQ-24 is a proxy answered questionnaire and will be used in individuals up to the age of 15. It consists of 24 questions of daily function, pain, pulmonary function and mobility [25]. EOSQ-24 will be collected from Swespine.
  o Oswestry Disability index (ODI). ODI is a back specific index measuring disability due to back pain [26]. It is the recommended instrument for studies concerning back pain. An index from 0–100 is calculated. An ODI of 0–20 indicate minimal disability, 21–40; moderate disability, 41–60; severe disability, 61–80; severely crippled, 81–100; bed-bound. ODI will be collected from Swespine.
  o EQ-5D 3 level is a generic quality of life instrument and consists of five areas reflecting mobility, self-care, usual activities, pain/discomfort and anxiety/depression [27]. Response alternatives range from no problems to extreme problems. An index can be calculated and depending on baseline value set used the index runs between approximately −0.5 (worst possible health) to 1.0 (best possible health). The EQ-5D-3L will be used for health economic analyses. The EQ-VAS is part of the EQ-5D and registers the patient’s self-rated health on a visual analogue scale (from 0 to 100; best). EQ-5D-3L will be collected from Swespine.
- Total procedure time as well as normalized to number of spinal levels from the upper to the lower instrumented vertebra.
- Intraoperative planning time (from start of planning in navigation software until last screw planned) (only when treated with ARSN or IRSN).
- Instrumentation time (total time for navigated/FH screw placement from first to last screw placement).
- Instrumentation time normalized to number of levels.
- Time for intraoperative verification imaging (CBCT and/or Fluoroscopy).
- Screw placement time (per screw, from owl to screw placed).
- Intraoperative blood loss.
- Intraoperative complications.
- Length of hospital stays.
- Administered doses of analgesia and types of analgesic medications required.
- Complications: at discharge, 30-, and 90-days.
- Readmissions and reasons for revision surgery: at 30-, 90-days, and 1-year.
- Costs per patient: at discharge, 30-, 90-days, and 1-year.

### Study follow-up

Study visits are shown in Table 1. Patients will be assessed before the surgery, during the surgery and during the inpatient stay, as well as postoperatively at 3 months, 1 year, and through the Swespine registry at 2, 5, and 10 years. There will be no additional visits for the patients involved in the study compared to the clinical routine.

**Table 1:** Study activities

	Visit 1: Screening Initial visit	Visit 2: Inpatient stay/surgery	Visits 3 + : 3 months +
Informed consent	X		
Demography	X		
Inclusion/exclusion criteria	X		
Randomization		X	
Outcome assessments	X	X	X*

^*^Outcomes assessed at regular clinical follow-ups; complications, standing spine radiographs. SRS-22r, EOSQ-24, ODI, EQ-5D-5L questionnaires collected as per Swespine routine on web or paper (preop, 1, 2, 5 and 10 years postoperatively)

Standard preoperative investigations include a low-dose computer tomography (CT), a whole spine standing radiograph and magnetic resonance imaging (MRI) of the spine. In case of navigation an intraoperative CBCT is performed. An intraoperative CBCT verification of implant positions is always performed in all cases of navigation and free hand procedures. In the immediate postoperative period, a low-dose CT is performed, as part of routine assessment. A whole spine standing radiograph is performed at 3 months and at 1 and 2 years. Curve size, type of scoliosis and other radiological parameters will be registered.

### Sample size calculation

#### Sample size for primary objective

Based on our own and studies from others we estimate that accuracy at 1 st attempt in any of the navigated groups is p_nav_ = 91% and accuracy in the free hand group is p_control_ 84% [14, 28]. Consequently, a one-sided test can be used. With a power of 80%, alpha = 0.05, a sample size of 301 screws is obtained using sample size calculation for chi-squared test with continuity correction with equal group sizes. With a possible loss of data of 10% we aim for 332 screws in each group (rounded up to the closest integer).

With a mean of 15 screws inserted in each patient [29], about 22 patients are needed in each group. A forward calculation demonstrates that for these sample sizes, and with the same accuracy as described above, a p-value of 0.006 will be returned.

#### Sample size for secondary objectives

Potentially the difference in intraoperative radiation could be two-fold when comparing navigated and free hand groups. The study will be sufficient to identify differences down to 20% between navigated and free hand when the navigated and free hand groups are pooled with a power of 80%, alpha = 0.05.

Minimal clinical important difference for SRS-22r is around 0.4. With a power of 80%, alpha = 0.05, a sample size of 37 patients in each group would be required to detect such a difference. Pooling data from the navigated and control groups will be done and sample size sufficient with an estimated 44 patients in the navigated and free hand groups, respectively.

Operative times will be compared between navigated and free hand groups. However, the current study sample size is underpowered to identify any differences in operating time when using our own earlier data with 403 (101) minutes and 361 (150) minutes in the navigated and free hand group respectively [14]; sample size needed would be 147 patients in each group. The data obtained in the current study may therefore only be indicative.

### Statistical analysis

In order to evaluate hypotheses of variables in contingency tables, the Chi-square or Fisher's Exact Tests will be used as appropriate. In order to test differences in continuous variables measured on at least interval scale between two independent groups the Student’s t-test for uncorrelated means will be used. The non-parametrical Mann–Whitney test will be used to test ordinal data. Regression analysis will be used to evaluate the dependency between variables and the Pearson correlation coefficient will be used in order to test independence between variables. In addition to that descriptive statistics will be used to characterize the data; means and 95% confidence intervals will be used for parametric data, medians and interquartile ranges for non-parametric data, and number (proportions) for categorical data. In case of multiple groups comparison. ANOVA, Kruskal–Wallis, and Chi-squared tests will be used respectively, along with post hoc analysis.

Protocol deviations will be recorded transparently. However, data from the different groups will be compared based on the'intention to treat'principle. An intention to treat (ITT) analysis means that all patients, regardless of treatment change, loss to follow-up or drop-out, remain in the analysis of the group to which they were randomized.

Statistical expertise will perform the statistical analysis.

Patient reported outcome measures consisting of ordinal scales for the different indices, will be compared using the Mann–Whitney test.

Data on total time on sick leave as well as the diagnosis used for sick leave will be collected.

Data on analgesic and antibiotic use will be collected from hospital files. Since different opioids may be used, conversion to morphine equivalents may be done. Data on other analgesics will be collected. Data on costs for the prescribed pharmaceuticals will be collected. Total amount of collected opioids (continuous data consisting of morphine equivalents) will be compared with the Student’s t-test or the Mann–Whitney test, depending on distribution.

Adverse events will be collected during the follow-up and classified as minor or major [30]. Data will be analyzed as dichotomous variables with Chi-square or Fisher tests.

Data on costs on the individual level will be collected. These consists of costs for the inpatient and outpatient visits (including surgery, radiographs, navigation), analgesic and antibiotic treatments, cost for sick leave. Working status at baseline and follow-ups will be queried.

Quality-adjusted life years (QALYs) will be calculated for each group (as measured by EQ-5D-5L). Combining cost and QALY yields incremental cost-effectiveness ratio (ICER) of the surgical intervention as compared to non-surgical care. To quantify the uncertainty around estimates, bootstrapping will be used. This allows the creation of the cost-effectiveness plane (a chart showing differences in outcomes and costs on each axis).

This ICER will then be compared to commonly used estimates of ICERs of other interventions (mainly surgeries and pharmaceutical interventions). Given the uncertainty around the official thresholds of willingness to pay for healthcare interventions, a cost-effectiveness acceptability curve will be drawn, showing the likelihood of the surgical intervention of being cost-effective given different levels of willingness to pay.

### Interim analyses

The study plan intends complete inclusion before analysis; therefore, an interim analysis is not planned.

### Data handling, quality, and ethics

#### Recording of data

Data will be collected from several sources (Table 2). Data primarily collected by Swespine will upon request from the research group be transferred into the study database.

**Table 2:** Collection of data

	Swespine	Hospital/patient files/case report form	Dosimeters	Cone beam CT/navigation equipment/regular CT
Patient reported outcome data	X			
Preoperative planning, neurophysiological monitoring		X		
Picture archiving and communications system (images)	X			
Data on planned and actual screw paths				X
Screening question answers	X			
Additional spine surgery	X			
Radiation to patient				X
Radiation to staff			X	
Analgesics/antibiotics used		X		
Number and extent of out- and inpatient visits	X	X		
Cost per patient		X		

#### Data storage and management

All data should be recorded, handled, and stored in a way that allows its accurate reporting, interpretation, and verification. All source data including informed consents, the completed study database, original protocol with amendments and the final report will be stored at the Karolinska University Hospital and Karolinska Institutet for a minimum period of 10 years after termination of the trial.

At the conclusion of the study, the occurrence of any protocol deviations will be determined. After these actions have been completed and the database has been declared to be complete and accurate, it will be locked and available for data analysis.

#### Quality control and quality assurance

The research coordinator will have regular contacts with the research team and participating subjects to confirm that the investigational team is adhering to the protocol. The investigators should ensure that all persons assisting with the trial are adequately informed and trained about the protocol and their trial related duties.

#### Audits and inspections

Authorized representatives of the Karolinska Trial Alliance, Karolinska University Hospital and Karolinska Institutet will perform audits and monitor the trial’s progress. The investigators must ensure that all study documents are accessible for auditing and inspection. The purpose of an audit or inspection is to examine all study-related activities and documents systematically and independently, to determine whether these activities were conducted, and data were recorded, analyzed, and accurately reported according to the protocol, and any applicable regulatory requirements.

#### Ethics

The study will be performed in accordance with the protocol, with the latest version of the Declaration of Helsinki, and applicable regulatory requirements. The Swedish national Ethical Review Agency has approved both protocols and the planned trial (DNR: 2021–03345, 2022–02882-02, 2023–05445-02).

The Principal Investigator is responsible for informing the Ethical Review Agency of any amendment to the protocol, in accordance with local requirements.

#### Informed consent

The investigators will ensure that the subject is given written information about the nature, purpose and possible risks and benefits of the study. Subjects must also be notified that they are free to discontinue from the study at any time. The subject should be given the opportunity to ask questions and allowed time to consider the information provided.

The subject’s signed and dated informed consent must be obtained before conducting any procedure specifically for the study.

The original, signed Informed Consent Form (ICF) must be stored at the study site.

### Subject data protection

The Informed Consent Form will incorporate wording that complies with relevant data protection and privacy legislation, and about the collection of data for the purposes of the study.

The Informed Consent Form will explain that study data will be collected from questionnaires, hospital files, images and health databases/registries and will be stored in a computer database, maintaining confidentiality in accordance with national data legislation.

### Insurances

The study subjects are covered by the Swedish Patient Injury Act.

### Protocol deviations and amendments

Modifications to the signed protocol are only possible through approved protocol amendments. Details of non-substantial amendments are to be clearly noted in the amended protocol.

In case of a substantial protocol amendment (e.g. change of; main purpose of the trial, primary/secondary variable, measurement of primary variable), the Ethical Review Agency must be informed and should be asked for its opinion/approval prior to implementation of amended protocol, as to whether a full re-evaluation of the ethical aspects of the study is necessary by the committee. This will be fully documented.

The Investigator will not implement any deviation from, or change to the protocol, without discussion with, and agreement by the study group and prior review and documented approval/favorable opinion of the amendment from the Ethical Review Agency, except where it is necessary to eliminate an immediate hazard to study subjects, or where the change(s) involves only logistical or administrative aspects of the study (e.g. change of telephone numbers).

### Report and publications

The trial was registered on clinicaltrial.gov prior to the start of inclusion (ID: NCT05107310). After completion of the study, the results will be analyzed, and a clinical study report will be prepared. Upon study completion and finalization of the study report the results of this trial will be disseminated through peer-reviewed publication and posted in a publicly accessible database of clinical trial results.

### Potential conflicts of interests

Financial support for the study was provided by Philips and Brainlab, with funding specifically allocated to the study center and not individual researchers. It is crucial to clarify that neither company played a role in the conceptualization and design of this trial. Additionally, neither entity will exert any influence over the investigative processes, monitoring, data retrieval, interpretations, or the writing and dissemination of research associated with this trial. AET was supported by Region Stockholm in a clinical research appointment. None of the researchers declare any personal conflict of interest.

## Discussion

We designed a randomized controlled trial, to compare free hand with navigation techniques in the placement of pedicle screws during spinal deformity surgery.

Despite a growing body of evidence endorsing the effectiveness of navigation techniques in the placement of pedicle screws, studies targeting the field of spinal deformity surgery are limited to small non-randomized and often retrospective series.

It is nonetheless in the field of deformity surgery where navigation technologies have demonstrated their most substantial impact on accuracy, particularly in neuromuscular scoliosis and the challenging terrain of the thoracic spine, where pedicles can be exceptionally small [31, 32]. Scoliosis may alter the anatomy of thoracic pedicles, posing a great challenge in spinal instrumentation [33].

To this day, retrospective studies reporting small differences in accuracy between FH and navigated techniques are often biased and lack robust matching protocols between cohorts. For instance, Noschenko et al. observed twice as many deformity cases in the navigation group compared to the freehand (FH) group [34]. This potentially explains the marginal difference in accuracy uncovered in that study, given the heightened complexity of deformity cases. Similarly, studies by Laudato et al. [35] and Shin et al. [36] demonstrated non-significant differences in accuracy but exhibited a larger proportion of screws in the thoracic spine in the navigation group compared to the FH group. Other prospective studies and recent meta-analyses of the literature have demonstrated similar results regarding the use of augmented-reality guidance [37, 38].

As a result, currently, the choice of techniques is mostly based on surgeon’s preference, and availability. This trial will be the first of its kind to address this topic in randomized and balanced cohorts of patients undergoing spinal deformity surgery with either navigation or FH techniques. Additionally, besides the planned trial, there are no studies addressing long-term quality of life outcomes in patients receiving pedicle screw placement with free hand versus navigation techniques.

Although intraoperative 3D imaging can increase the accuracy of pedicle screw placement, this technique may increase the total radiation exposure to staff and patients compared to standard fluoroscopy. We therefore aimed to study radiation exposure to both patients and surgical staff.

In summary, this trial intends to provide high levels of evidence on the topic surrounding the use of different types of navigation techniques for pedicle screw placement in spine deformity surgery.

### Limitations

This trial has several limitations including the lack of a triple-blinded design, and the relatively small sample size which does not allow for full interpretation of secondary outcomes. In that aspect, although blinding does not affect any of the primary outcomes of this trial, it may potentially influence the secondary outcomes, especially PROMs, which constitutes a noteworthy limitation. Also, the trial will be conducted in a single country and at a single center, with surgeries being performed by highly experienced spine surgeons, which hampers generalizability of the findings.

## Data Availability

No datasets were generated or analysed during the current study.

## References

[1] Tsirikos AI, Roberts SB, Bhatti E. Incidence of spinal deformity surgery in a national health service from 2005 to 2018. Bone Jt Open. 2020;1:19–28. 33215103 10.1302/2633-1462.13.BJO-2020-0001.R1 PMC7659651

[2] Beschloss A, Dicindio C, Lombardi J, Varthi A, Ozturk A, Lehman R, et al. Marked increase in Spinal Deformity Surgery throughout the United States. Spine (Phila Pa 1976). 2021;46:1402–8. 10.1097/BRS.0000000000004041 33769412

[3] Koshimizu H, Nakashima H, Ohara T, Tauchi R, Kanemura T, Shinjo R, et al. Implant-related complications after spinal fusion: A multicenter study. Global Spine J. 2024;14:74–81. 35400240 10.1177/21925682221094267 PMC10676178

[4] Gharios M, El-Hajj VG, Frisk H, Ohlsson M, Omar A, Edström E, et al. The use of hybrid operating rooms in neurosurgery, advantages, disadvantages, and future perspectives: a systematic review. Acta Neurochir (Wien). 2023;165:2343–58. 37584860 10.1007/s00701-023-05756-7 PMC10477240

[5] Fichtner J, Hofmann N, Rienmüller A, Buchmann N, Gempt J, Kirschke JS, et al. Revision rate of misplaced pedicle screws of the thoracolumbar spine–comparison of three-dimensional fluoroscopy navigation with freehand placement: A systematic analysis and review of the literature. World Neurosurg. 2018;109:e24-32. 28951183 10.1016/j.wneu.2017.09.091

[6] Staartjes VE, Klukowska AM, Schröder ML. Pedicle screw revision in robot-guided, navigated, and freehand thoracolumbar instrumentation: A systematic review and meta-analysis. World Neurosurg. 2018;116:433-443.e8. 29859354 10.1016/j.wneu.2018.05.159

[7] Liu Z, Jin M, Qiu Y, Yan H, Han X, Zhu Z. The superiority of intraoperative O-arm navigation-assisted surgery in instrumenting extremely small thoracic pedicles of adolescent idiopathic scoliosis. Medicine (Baltimore). 2016;95: e3581. 27149486 10.1097/MD.0000000000003581 PMC4863803

[8] Jin M, Liu Z, Qiu Y, Yan H, Han X, Zhu Z. Incidence and risk factors for the misplacement of pedicle screws in scoliosis surgery assisted by O-arm navigation—analysis of a large series of one thousand, one hundred and forty five screws. Int Orthop. 2017;41:773–80. 27999927 10.1007/s00264-016-3353-6

[9] Iop A, El-Hajj VG, Gharios M, de Giorgio A, Monetti FM, Edström E, et al. Extended reality in neurosurgical education: A systematic review. Sensors (Basel). 2022;22:6067. 36015828 10.3390/s22166067 PMC9414210

[10] Burström G, Nachabe R, Persson O, Edström E, Elmi Terander A. Augmented and virtual reality instrument tracking for minimally invasive spine surgery. Spine (Phila Pa 1976). 2019;44:1097–104. 10.1097/BRS.0000000000003006 30830046

[11] Elmi-Terander A, Skulason H, Söderman M, Racadio J, Homan R, Babic D, et al. Surgical navigation technology based on augmented reality and integrated 3D intraoperative imaging. Spine (Phila Pa 1976). 2016;41:E1303–11. 10.1097/BRS.0000000000001830 PMC5113235 27513166

[12] Elmi-Terander A, Burström G, Nachabe R, Skulason H, Pedersen K, Fagerlund M, et al. Pedicle screw placement using augmented reality surgical navigation with intraoperative 3D imaging. Spine (Phila Pa 1976). 2019;44:517–25. 10.1097/BRS.0000000000002876 PMC6426349 30234816

[13] Ghaednia H, Fourman MS, Lans A, Detels K, Dijkstra H, Lloyd S, et al. Augmented and virtual reality in spine surgery, current applications and future potentials. Spine J. 2021;21:1617–25. 33774210 10.1016/j.spinee.2021.03.018

[14] Elmi-Terander A, Burström G, Nachabé R, Fagerlund M, Ståhl F, Charalampidis A, et al. Augmented reality navigation with intraoperative 3D imaging vs fluoroscopy-assisted free-hand surgery for spine fixation surgery: a matched-control study comparing accuracy. Sci Rep. 2020;10. 10.1038/s41598-020-57693-5 PMC6971085 31959895

[15] Urbanski W, Jurasz W, Wolanczyk M, Kulej M, Morasiewicz P, Dragan SL, et al. Increased radiation but no benefits in pedicle screw accuracy with navigation versus a freehand technique in scoliosis surgery. Clin Orthop Relat Res. 2018;476:1020–7. 29432262 10.1007/s11999.0000000000000204 PMC5916595

[16] Good CR, Orosz L, Schroerlucke SR, Cannestra A, Lim JY, Hsu VW, et al. Complications and revision rates in minimally invasive robotic-guided versus fluoroscopic-guided spinal fusions: The MIS ReFRESH prospective comparative study. Spine (Phila Pa 1976). 2021;46:1661–8. 10.1097/BRS.0000000000004048 PMC8565511 33826591

[17] Staartjes VE, Molliqaj G, van Kampen PM, Eversdijk HAJ, Amelot A, Bettag C, et al. The European Robotic Spinal Instrumentation (EUROSPIN) study: protocol for a multicentre prospective observational study of pedicle screw revision surgery after robot-guided, navigated and freehand thoracolumbar spinal fusion. BMJ Open. 2019;9:e030389. 31501123 10.1136/bmjopen-2019-030389 PMC6738706

[18] Edström E, Burström G, Nachabe R, Gerdhem P, Elmi TA. A novel augmented-reality-based surgical navigation system for spine surgery in a hybrid operating room: Design, workflow, and clinical applications. Oper Neurosurg (Hagerstown). 2020;18:496–502. 31504859 10.1093/ons/opz236

[19] Burström G, Buerger C, Hoppenbrouwers J, Nachabe R, Lorenz C, Babic D, et al. Machine learning for automated 3-dimensional segmentation of the spine and suggested placement of pedicle screws based on intraoperative cone-beam computer tomography. J Neurosurg Spine. 2019;31:147–54. 30901757 10.3171/2018.12.SPINE181397

[20] Edström E, Burström G, Omar A, Nachabe R, Söderman M, Persson O, et al. Augmented reality surgical navigation in spine surgery to minimize staff radiation exposure. Spine (Phila Pa 1976). 2020;45:E45–53. 10.1097/BRS.0000000000003197 31415457

[21] Burström G, Cewe P, Charalampidis A, Nachabe R, Söderman M, Gerdhem P, et al. Intraoperative cone beam computed tomography is as reliable as conventional computed tomography for identification of pedicle screw breach in thoracolumbar spine surgery. Eur Radiol. 2021;31:2349–56. 33006659 10.1007/s00330-020-07315-5 PMC7979653

[22] Frisk et al. Automatic image registration on intraoperative CBCT compared to surface matching registration on preoperative CT for spinal navigation: accuracy and workflow. International Journal of Computer Assisted Radiology and Surgery. 2024. 10.1007/s11548-024-03076-4 PMC10973038 38378987

[23] Gertzbein SD, Robbins SE. Accuracy of pedicular screw placement in vivo. Spine (Phila Pa 1976). 1990;15:11–4. 10.1097/00007632-199001000-00004 2326693

[24] Danielsson AJ, Romberg K. Reliability and validity of the Swedish version of the scoliosis research society–22 (SRS-22r) patient questionnaire for idiopathic scoliosis. Spine (Phila Pa 1976). 2013;38:1875–84. 10.1097/BRS.0b013e3182a211c0 23846501

[25] Matsumoto H, Williams B, Park HY, Yoshimachi JY, Roye BD, Roye DP, et al. The final 24-item early onset scoliosis questionnaires (EOSQ-24): Validity, reliability and responsiveness. J Pediatr Orthop. 2018;38:144–51. 27299779 10.1097/BPO.0000000000000799 PMC5562528

[26] van Hooff ML, Spruit M, Fairbank JCT, van Limbeek J, Jacobs WCH. The Oswestry disability index (version 2.1a). Spine (Phila Pa 1976). 2015;40:E83–90. 10.1097/BRS.0000000000000683 25575092

[27] Burström K, Johannesson M, Diderichsen F. Swedish population health-related quality of life results using the EQ-5D. Qual Life Res. 2001;10:621–35. 11822795 10.1023/a:1013171831202

[28] Baky FJ, Milbrandt T, Echternacht S, Stans AA, Shaughnessy WJ, Larson AN. Intraoperative computed tomography–guided navigation for pediatric spine patients reduced return to operating room for screw malposition compared with freehand/fluoroscopic techniques. Spine Deform. 2019;7:577–81. 31202374 10.1016/j.jspd.2018.11.012 PMC6578871

[29] Charalampidis A, Möller A, Wretling M-L, Brismar T, Gerdhem P. Implant density is not related to patient-reported outcome in the surgical treatment of patients with idiopathic scoliosis. Bone Joint J. 2018;100-B:1080–6. 10.1302/0301-620X.100B8.BJJ-2017-1114.R1 30062942

[30] Ibañez FAL, Hem S, Ajler P, Vecchi E, Ciraolo C, Baccanelli M, et al. A new classification of complications in neurosurgery. World Neurosurg. 2011;75:709–15. 21704941 10.1016/j.wneu.2010.11.010

[31] Jin M, Liu Z, Liu X, Yan H, Han X, Qiu Y, et al. Does intraoperative navigation improve the accuracy of pedicle screw placement in the apical region of dystrophic scoliosis secondary to neurofibromatosis type I: comparison between O-arm navigation and free-hand technique. Eur Spine J. 2016;25:1729–37. 25967559 10.1007/s00586-015-4012-0

[32] Liu H, Chen W, Liu T, Meng B, Yang H. Accuracy of pedicle screw placement based on preoperative computed tomography versus intraoperative data set acquisition for spinal navigation system. J Orthop Surg (Hong Kong). 2017;25:230949901771890. 10.1177/2309499017718901 28673199

[33] Akazawa T, Kotani T, Sakuma T, Minami S, Tsukamoto S, Ishige M. Evaluation of pedicle screw placement by pedicle channel grade in adolescent idiopathic scoliosis: should we challenge narrow pedicles? J Orthop Sci. 2015;20:818–22. 26124077 10.1007/s00776-015-0746-0

[34] Noshchenko A, Cain CMJ, Zaghloul K, Lindley EM, Kleck C, Burger EL, et al. Pedicle screw placement assisted by 3D imaging (O-arm system with StealthStation® software) versus free-hand technique for multilevel posterior thoracolumbar fusion. Curr Orthop Pract. 2018;29:151–6.

[35] Laudato PA, Pierzchala K, Schizas C. Pedicle screw insertion accuracy using O-arm, robotic guidance, or freehand technique. Spine (Phila Pa 1976). 2018;43:E373–8. 10.1097/BRS.0000000000002449 29019807

[36] Shin M-H, Ryu K-S, Park C-K. Accuracy and safety in pedicle screw placement in the thoracic and lumbar spines : Comparison study between conventional C-arm fluoroscopy and navigation coupled with O-arm® guided methods. J Korean Neurosurg Soc. 2012;52:204. 23115662 10.3340/jkns.2012.52.3.204 PMC3483320

[37] Youssef S, McDonnell JM, Wilson KV, Turley L, Cunniffe G, Morris S, et al. Accuracy of augmented reality-assisted pedicle screw placement: a systematic review. Eur Spine J. 2024;33:974–84. 38177834 10.1007/s00586-023-08094-5

[38] Altorfer FCS, Kelly MJ, Avrumova F, Burkhard MD, Zhu J, Abel F, et al. Pedicle screw placement with augmented reality versus Robotic-assisted surgery. Spine (Phila Pa 1976). 2024. 10.1097/BRS.0000000000005147. 10.1097/BRS.0000000000005147 39231739

